# Situating Language in the Real-World: The Role of Multimodal Iconicity and Indexicality

**DOI:** 10.5334/joc.113

**Published:** 2021-08-23

**Authors:** Margherita Murgiano, Yasamin Motamedi, Gabriella Vigliocco

**Affiliations:** 1 Experimental Psychology, University College London, GB

**Keywords:** iconicity, multimodal communication, language acquisition, language processing

## Abstract

In the last decade, a growing body of work has convincingly demonstrated that languages embed a certain degree of non-arbitrariness (mostly in the form of iconicity, namely the presence of imagistic links between linguistic form and meaning). Most of this previous work has been limited to assessing the degree (and role) of non-arbitrariness in the speech (for spoken languages) or manual components of signs (for sign languages). When approached in this way, non-arbitrariness is acknowledged but still considered to have little presence and purpose, showing a diachronic movement towards more arbitrary forms. However, this perspective is limited as it does not take into account the situated nature of language use in face-to-face interactions, where language comprises categorical components of speech and signs, but also multimodal cues such as prosody, gestures, eye gaze etc. We review work concerning the role of context-dependent iconic and indexical cues in language acquisition and processing to demonstrate the pervasiveness of non-arbitrary multimodal cues in language use and we discuss their function. We then move to argue that the online omnipresence of multimodal non-arbitrary cues supports children and adults in dynamically developing situational models.

## 1. Introduction

Moving away from a more traditional view of language (e.g., de Saussure, 1916; Hockett, 1960), numerous studies in recent years have focused on non-arbitrary features of language such as iconicity (i.e., the presence of imagistic links between some features of the linguistic form and attributes of the corresponding referent) in both spoken and sign languages (see for example Dingemanse et al., 2015; Perniss et al., 2010 for reviews), and the indexicality (i.e., signs that index, point to, specific referents) of certain signs like pronouns in sign languages (e.g., the sign for the pronoun “you” in many sign languages consists in an extended index finger just like in pointing toward the person) (Liddell, 2003; Johnston, 2013; Meier & Lillo-Martin, 2013).

However, human languages are still thought to be primarily arbitrary, with properties like iconicity and indexicality considered as marginal (Dingemanse, 2018). Non-arbitrariness is still generally argued to be a negligible feature, uncommon in the majority of studied linguistic systems — particularly English. Even in sign languages, where non-arbitrariness is indisputably more visible, it has been hypothesised to reduce over time in favour of more arbitrary forms (Emmorey, 2014; Frishberg, 1975; Klima & Bellugi, 1979) or in any case not to impact language learning or use (e.g., Pettito, 1987). This paper offers a different perspective on the topic, a perspective that we believe has far reaching consequences not only for how we characterise the presence, function and limits of non-arbitrariness in language, but language itself.

Previous work investigating non-arbitrariness has invariably focussed on language **as a population-level system** in which the structured, categorical components of speech (for spoken languages) and manual signs (for sign languages) can be described in a population at a given time point, to characterise the language synchronically and track diachronic change. In this way, language has been empirically studied as speech/sign or text, separately from other contextual components of communication. While this perspective has been undeniably fruitful, we believe it can only lead to a partial understanding of language and its features because it offers a distilled abstraction of how language really manifests.

Linguistic use is first of all inevitably embedded in a *physical context*, the environment in the here and now which can disambiguate the message or offer useful resources to enhance its communicative power (Clark, 2016; Mondada, 2019). Most importantly, while language certainly exists as the categorical components of speech and signs, these components are never alone in the situated *communicative context* where language is learnt and used: online, face-to-face interactions present a composite and richer message exploiting simultaneously multiple articulators and channels. Other cues, such as gesture, prosody and eye gaze (to name a few) are always present, have been argued to be essential for the phylogenetic and ontogenetic emergence of language, and are key components of meaning-making (Holler & Levinson, 2019; Kendon, 2012, 2014; Perniss, 2018; Vigliocco et al., 2014).

In this paper, along with other scholars (e.g., Hasson et al., 2018; Holler & Levinson, 2019), we argue that we should broaden our lens beyond a notion of language as something that can be investigated in a single communicative channel (e.g., vocal *or* manual) to the study of **language as situated**, namely, as the ensemble of speech (or sign) in specific communicative and physical contexts as dynamically presenting during communicative interactions. This perspective forces a rethinking of the traditional distinction between what we consider as linguistic and non-linguistic (Fontana, 2008; Kendon, 2012, 2014; Slobin, 2008). Similarly, it pushes us to rethink what we consider a ‘core’ feature of language and what is instead a secondary or even negligible attribute. Thus, when looking at *language as a system*, non-arbitrariness in the linguistic form appears to be a marginal feature that decreases over time as the result of pressures to (for example) reduce production effort and memory demands.

However, when looking at *language as situated*, the presence of non-arbitrariness is much more than marginal: during face-to-face interactions language users draw from both linguistic resources available in the system and other online multimodal resources such as iconic gestures and iconic prosody (Drijvers & Özyürek, 2017; Herold et al., 2012; Iverson et al., 1999; Vigliocco et al., 2019) as well as points and eye gaze (Cooperrider, 2016; Holler et al., 2014; Motamedi et al., 2020). Taken together, these iconic and indexical multimodal cues provide effective mechanisms to single out and bring “to the mind’s eye” referents being talked about.

Note here that we distinguish iconicity and indexicality as non-arbitrary components that have been undervalued under the language as a system approach. Another component of human language that is often included along with iconicity and indexicality when discussing non-arbitrariness is systematicity, which refers to regular correspondences between form and meaning, without the form having to represent the meaning through resemblance or analogy (Dingemanse et al., 2015; Monaghan et al., 2014). However, we suggest that systematicity is qualitatively different from both iconicity and indexicality. Where iconicity and indexicality can exist independently from a system (i.e., a spontaneous pointing gesture can be understood without reference to a pointing system), systematic correspondences between form and meaning can only be understood in relation to the whole system. As such, systematicity is best understood primarily under the language as a system view, and we therefore focus on iconicity and indexicality as revealed through the language as situated perspective.

Below, we first briefly review research on non-arbitrariness carried out in the language as a system tradition, spelling out the main shortcomings of such an approach. We then introduce the perspective of language as situated by outlining how language, as it is used in face-to-face, interactive settings, is dynamic, multimodal and contextualised. We discuss the implications of this approach for our understanding of iconicity and indexicality in two research domains— language acquisition and language processing— as examples of areas where considering language only at the system level is especially problematic because both are for the most part carried out in face-to-face contexts where all the multimodal cues are available. In both cases, considering language as only speech or only sign leaves us with an impoverished view of how humans learn and process language.

## 2. Non-Arbitrariness from the language as a system perspective

Research on iconicity has seen a boom over the last decade, with an increasing acknowledgement of iconicity as a non-trivial property of language (Perniss et al., 2010), present in both signed and spoken languages (Dingemanse, 2018; Dingemanse et al., 2015; Perniss et al., 2010). As already mentioned, most investigations have focussed on languages as structural, rule-based systems of form-meaning mappings, reflecting population-level biases for ease of articulation, ease of learning and communicative efficiency. Thus, research first has established the presence of iconicity at different levels of description. At the lexical level, whole words or signs are judged to be iconic (i.e., ‘moo’ sounds like the noise a cow makes, or the British Sign Language (BSL) sign for BOOK represents the leaves of a book; Boyes-Braem, 1986; Perry et al., 2015; Pietrandrea, 2002; Vinson et al., 2008; Winter et al., 2017). At the sub-lexical level, meaningful correspondences exist between particular phonemes or certain acoustic properties and particular semantic properties, such as the vowel *i*: being associated with small size in spoken languages (Knoeferle et al., 2017; Marchand, 1959). Such correspondences also exist in sign languages, with phonological parameters such as movement, location or handshape being iconically linked to the meaning of the sign (Boyes-Braem, 1981; Brentari, 2012; Emmorey & Herzig, 2003; Thompson et al., 2010). Finally, iconicity has been studied at the syntactic level, where, for example, the structure of the signed or spoken phrase can represent the structure of the event in both spoken and sign languages (Christensen et al., 2016; Diessel, 2008; Strickland et al., 2015; Wilbur, 2004).

In addition to having confirmed that iconicity is present across languages, research has established that iconicity plays a role in language learning and processing. For instance, iconicity has been shown to be common in children’s early vocabularies (Caselli & Pyers, 2019; Perry et al., 2017; Tardif et al., 2008; D. P. Vinson et al., 2008) and argued to facilitate word and sentence-level comprehension in toddlers and young children (De Ruiter et al., 2018; Imai et al., 2008). In adult language users, studies have shown that adults presented with sound-symbolic words from unfamiliar languages (e.g., Japanese, Semai) can guess their meanings above what would be expected by chance (Dingemanse et al., 2016; Lockwood et al., 2016). Though the role of iconicity in lexical acquisition is still the subject of a lively debate (see for example Ortega, 2017 for a review), most recent research has accumulated evidence suggesting that iconicity helps to ground referential communication providing information about properties of real-world referents that may be of particular help for young language learners (Imai & Kita, 2014) and for users and learners of emerging linguistic systems (Fay et al., 2013; Perlman et al., 2015; Roberts et al., 2015).

Indexicality, in contrast, is largely ignored by the language as a system approach. At the lexical level, demonstrative pronouns have been considered indexical, such that they index a meaning without using its conventional lexical form. However, this differs somewhat from our definition of indexical as providing a visual link to the intended referent (e.g., through a finger point). Pointing, and other forms of deixis have largely been ignored from a systemic point of view for spoken languages. In sign language research, indexical points have long been understood as part of the grammatical systems of most sign languages. As a consequence, they are often studied under a language as a system view, that posits grammatical pointing as qualitatively different from gestural points. As such, the overlap between gestural and grammatical points has been argued not to be understandable by children. Pettito (1987) argues that the finding that children learning ASL can erroneously produce “you” (point toward you) when “I” (point toward me) is intended, just like children speaking English, provides evidence for the independence of grammatical and gestural systems. More recently, that perspective has shifted, to understand the similarities between grammatical and gestural pointing (Cormier et al., 2013), where grammatical points, though very similar to gestural points, are systematically constrained by other aspects of the linguistic system (Fenlon et al., 2019).

Understanding the effects of non-arbitrariness on language learning also highlights what non-arbitrariness *cannot* do. For example, from a system-wide perspective, iconicity becomes limiting when there is an asymmetry between the dimensions of the meaning space and the dimensions of the signal space (Gasser, 2004; Little et al., 2017): i.e., as a language grows in the number of meanings it wants to refer to, it becomes more difficult to maintain iconicity across the system. Furthermore, iconicity purportedly hinders discriminability in crowded meaning spaces, such that lexical items that occur in dense semantic networks tend to be less iconic (Sidhu & Pexman, 2018), and the specified nature of iconic forms, referring to particular properties of referents, may mean that iconic forms are less well-suited to refer to more general or more abstract concepts (Lupyan & Winter, 2018). These observations suggest that from the systemic perspective, iconicity may serve little purpose beyond first language acquisition, and beyond the initial grounding and early evolution of novel referential systems. That is, in mature languages and speakers, the remaining iconic forms are ‘relics’ of our (phylogenetic and ontogenetic) history. This hypothesis has some support from natural language data. Studies of children’s early vocabulary and from child-directed language, based on English-speaking populations, suggest that the proportion of iconic words reduce in both cases as children get older (Laing, 2014; Perry et al., 2017; Vigliocco et al., 2019). For understanding the evolution of iconic forms, Frishberg’s (1975) study of signs from American Sign Language (ASL) documented a movement from more iconic to more abstract forms, a pattern supported by results from experimental studies of novel communication systems (Garrod et al., 2007; Theisen et al., 2010).

Yet, there are at least some studies that have shown processing effects of iconicity in adult language users. First, there is evidence that adult speakers can identify correspondences between sounds and meanings (e.g., Köhler, 1929; Ramachandran & Hubbard, 2001). For example it has been shown that Dutch and English speakers can guess the meaning of sound-symbolic Japanese words above chance (Lockwood et al., 2016; Oda, 2000) and English speakers have been shown to map Japanese sound-related ideophones to similar meanings as Japanese native speakers (Iwasaki et al., 2007). Lexical iconicity has also been shown to facilitate processing in lexical decision tasks; for example, Sidhu et al. (2020) found that iconic words were processed more quickly in a visual lexical decision tasks by healthy English-speaking adults, and Meteyard et al. (2015) found a facilitation effect in patients with aphasia in an auditory lexical decision task, with participants recognising iconic words faster than non-iconic ones. In sign language, Vinson et al (2015) showed that iconic signs in BSL are produced faster than less-iconic ones. These, however, are rather limited effects.

As already introduced, there are several important shortcomings to the system-level view of language that may be critical for our discussion. First, work in this tradition is shaped by the languages from western-industrialised communities (especially English) on which it is based, and which have more limited sound-symbolic and iconic vocabularies than other linguistic systems, such as non-Indo-European languages and sign languages (Dingemanse, 2018; Vigliocco et al., 2014). As such, even within the system-level view, the prevalence of non-arbitrariness across the world’s languages may have been underestimated. Second, this view fails to account for the very systematic and dynamic nature of language behaviours it aims to capture. By focusing on the static properties of language (e.g., either lexical *or* phonological *or* syntactic iconicity), we do not account for the multiplex ways in which behaviours associated with language use interact. For example, evidence showing that the proportion of iconic words in children’s vocabularies declines with age may support the view that iconicity is less useful beyond early word learning, but little to no data exists that tracks the use of non-arbitrary forms in other modalities (such as iconic and pointing gestures), or how children might draw from information in the lexical and gestural channels simultaneously.

Consequently, we propose that a more ecological model of language use, one that accounts for face-to-face interaction and multimodal behaviour, is imperative to gain a more comprehensive understanding of the role that non-arbitrariness plays in both language learning and processing. Furthermore, given that the human language capacity likely evolved from pre-linguistic communicative interactions (Levinson & Holler, 2014), an understanding of the contextual constraints of language will further shed light on the ways in which the structures and components of language are adapted for learning and use in interaction (Chater & Christianesen, 2008; Kirby et al., 2015).

Importantly, the narrow focus of the language as a system approach is not only an issue for research on non-arbitrariness, but cognitive science in general. Indeed, there is increasing call among language scientists for more ecologically valid and multiplex approaches, such as those concerning language processing (Holler & Levinson, 2019), neurobiology (Hasson et al., 2018) and language development (Rączaszek-Leonardi et al., 2018). In the following sections, we use non-arbitrariness to highlight how a focus on language as a stable and static rule-based system fails to account for the rich set of non-arbitrary behaviours that govern natural language learning and use, and suggest that understanding the contextual constraints and affordances of face-to-face interaction can offer a more comprehensive picture.

## 3. Non-Arbitrariness from the language as situated perspective

Within linguistics, pragmatics has investigated language use in context, showing that the access to a message doesn’t solely rely on the knowledge of underlying lexical and grammatical rules, but on a larger range of situational factors. Among these factors shown to affect language comprehension are the cultural assumptions and habits shaping a shared ‘common ground’ between speaker and listener (Bohn & Köymen, 2018; Clark, 1996) and socio-pragmatic skills, e.g., the ability to infer communicative intentionality (Grice, 1975). This approach has revealed language as a joint practice with which interlocutors cooperatively signify reality (Clark, 1996; Tomasello, 2008) and act on it, for example influencing others’ behavior – like asking a colleague to close the window just by saying “it’s very cold in the office today!” – or performing proper acts, like when we promise, demand or forbid something just by saying so (Austin, 1962; Searle, 1969).

However, shedding light on the contextual nature of linguistic comprehension also means acknowledging that language use is, for the most part, embedded in face-to-face interactions where language manifests through the combination of speech and signs and other simultaneous embodied resources that allow us to convey meanings. Levinson and Holler (2014) argue that human language is the result of an evolutionary process originated in situated communicative interactions that have led it to evolve as a ‘system of systems’ where various expressive channels developed phylogenetically to contribute with their different strengths to the communicative goal. We observe this multi-layered nature of language everyday, engaging simultaneously the audio-vocal, the visual-gestural and the oro-facial channel, and with listeners and learners in both spoken and sign languages extracting information from a multimodal communicative context. In spoken languages, the linear speech component is combined with concurrent mouth movements that influence speech perception (Chandrasekaran et al., 2009; Mcgurk & Macdonald, 1976) and prosodic modulations which mark information in beneficial ways (Cutler et al., 1986, 1997; Shintel et al., 2014). Gestures naturally co-occurring with speech have been largely recognized as an integral part of the message (Abner et al., 2015; Kendon, 2004; McNeill, 1992), along with other cues such as eye gaze (Staudte & Crocker, 2011), as well as facial expressions and body movements. Brow movements, facial expressions and posture shifts have grammatical and lexical functions in the majority of sign languages (Mohr, 2014; Sandler, 1999), and are in some cases necessary to distinguish between meanings (Pfau & Quer, 2010; Woll, 2014). Finally, when using language we also frequently resort to the surrounding physical context, for example manipulating objects to demonstrate how they work or using them as props to represent something else (Clark, 2016; Mondada, 2019). In this way, object-directed actions are frequently co-opted in communication to offer visual information about the message (Alibali et al., 2011; Brand et al., 2002, 2007, S. Kelly et al., 2015; Vigliocco, Motamedi, et al., 2019).

In short, language used in face-to-face online communication is a multimodal phenomenon (Kendon, 2014; Perniss, 2018; Vigliocco et al., 2014) enacted through the combination of different resources – speech/signs, body gestures and object manipulations – the use of which is pervasive and, we argue, advantageous to comprehension and learning. In the language as situated framework, these features are not defined negatively (e.g., “non-linguistic” signals, “non-manual” components), but are instead conceived as part and parcel of language (Kendon, 2012, 2014; Liddell, 2003; Perniss, 2018; Slobin, 2008; Vigliocco et al., 2014). For this reason, the language as a system view is not incompatible with the perspective proposed here: rather, the latter includes the former, with language as a structure of categorical components being part of a broader, diversified ensemble that constitutes language use situated in the communicative and physical context.

**Figure 1:**
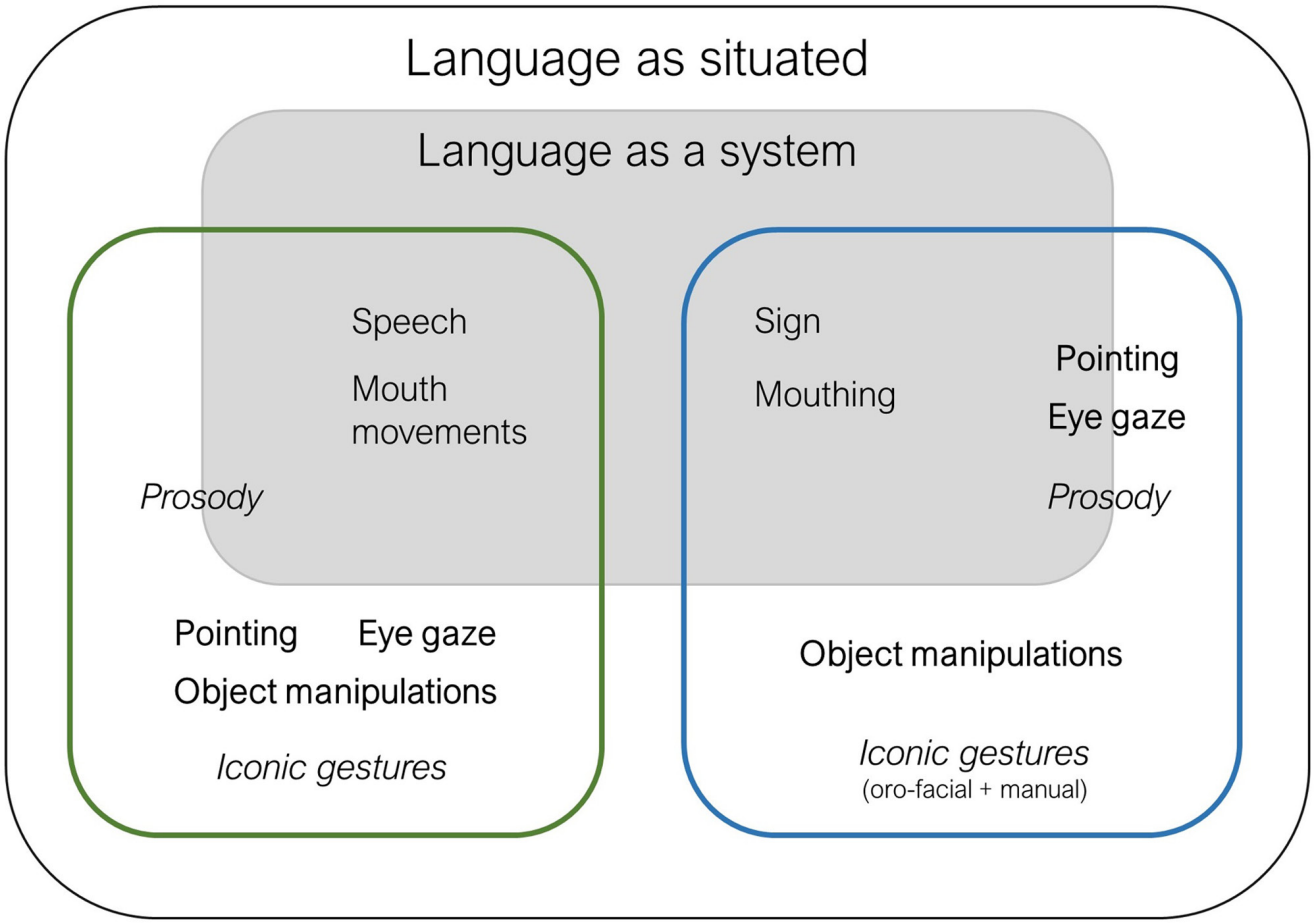
Components of language under a language as situated perspective. Language as a system remains in place under this view, containing those behaviours characterised as systemic. We show different communicative behaviours for both signed (blue square) and spoken (green square) languages. Iconic cues are shown in italics, indexical cues in bold. Some features (e.g., pointing in sign languages) can be characterised as either systemic or contextual.

If we take the language as situated view, we are necessarily pushed to questioning the dogma of language as primarily arbitrary. The multimodal components pervasively accompanying linguistic exchanges often provide non-arbitrary relations to the meaning in the form of both iconicity — exhibiting the qualitative features of the referent in the communicative form — and indexicality — creating an associative visual link with the referent (Peirce, 1974). Iconic mappings, while available — as we pointed out — in the linguistic repertoire at different levels, can be also exploited *in-situ* (Holler & Levinson, 2019). For example, spoken languages exploit iconic gestures performed with the hands (Kendon, 2008; McNeill, 1992) or the whole body (Clark, 2016; Stec et al., 2016), as well as prosodic modulations such as slowing the speech rate to refer to a slow action (Nygaard et al., 2009). Sign languages exhibit similar prosodic modification of signs; e.g., slower motion to indicate an effortful action (Perniss, 2018) as well as channel-specific phenomena such as role-taking; e.g., a narrator shifting to the viewpoint of the actor (Cormier et al., 2015). Furthermore, eye gaze movements following the position of an object, object manipulations or pointing gestures can be used to index referents in both sign and spoken languages. We illustrate the range of cues we discuss here under the language as situated framework in Figure 1.

We suggest that looking at language as situated provides a more ecologically valid framework necessary to fully understand how the different components of language are employed during face-to-face interactions in the online physical and communicative context. This view reveals human communication as a rich set of arbitrary and non-arbitrary components used in context-sensitive ways to fulfill different communicative goals. In particular, it underscores non-arbitrary communicative mechanisms in the form of indexical and iconic multimodal cues as pervasively exploited by language users to reinforce the denotative function of the message and enhance its depictive power (Clark, 1996, 2016; Sallandre & Cuxac, 2001), allowing interlocutors to converge on a situational model. Below, we review how caregivers and children exploit non-arbitrary multimodal cues to fulfill these functions in situated communication in ways that support language acquisition.

## 4. Non-Arbitrariness in Language Acquisition

Over the years, research has highlighted the dialogical and embodied nature of language development, stressing the fundamental role played by contextual cues (Bohn & Frank, 2019; Rączaszek-Leonardi et al., 2018). For example, a study by Cartmill et al. (2013) illustrated that referential transparency (operationalized as how clearly the meaning of words can be inferred from accompanying contextual cues) is an important predictor of vocabulary size at a later age (54 months). The physical context in which children learn provides critical cues, such as the objects being referred to and the affordances for actions being talked about. However, the communicative context also provides a wealth of useful cues, many of which afford iconic and indexical strategies.

Caregivers use iconicity in interactions with their children: they consistently use prosodic modulations to facilitate the interpretation of contrasting meanings (Herold, Nygaard, & Namy, 2011). Furthermore, when asked to produce novel adjectives, adults using child-directed language rely on systematic sound-to-meaning correspondences (Nygaard et al., 2009) that preschool children successfully use to infer meaning when no other cues are present (Herold, Nygaard, Chicos, et al., 2011). Manual iconicity has also been found to support learning: hearing two-and-a-half-year olds have been shown to be able to recognize iconic mappings embedded in unfamiliar signs representing actions (Tolar et al., 2008) and to learn iconic gestures at 26 months, when they exhibit difficulties in learning arbitrary gestural labels (Namy et al., 2004). Furthermore, iconic co-speech gestures have been shown to facilitate verb learning in children as young as 2 (Goodrich & Hudson Kam, 2009) and to influence, with their depictive features, the generalizations of ambiguous novel labels in 3-year olds (Mumford & Kita, 2014).

Caregivers also use indexical cues. Caregiver eye gaze has been shown to work as an attention-getter (Senju & Csibra, 2008) that children seem to be able to use early on in development (D’Entremont, 2000; Hofsten et al., 2005; Senju & Csibra, 2008). Points are the most common gesture used by caregivers very early on, and it has been found that their production in association with the label correlates with children’s vocabulary development (Iverson et al., 1999; O’Neill et al., 2005; Rowe & Goldin-Meadow, 2009). Object manipulations have also been considered to provide a useful cue: caregivers have been found to exaggerate their hand actions when focusing on new objects, enhancing features like repetitiveness and range of motion in ways thought to facilitate children’s attention and learning (Brand et al., 2002, 2007).

Considering iconicity and indexicality from the language as situated perspective allows us to go beyond asking whether non-arbitrariness in caregivers’ input may or not support learning to asking questions concerning under which conditions non-arbitrariness will be most useful to children. Most of the work described above has considered lexical acquisition in situations in which referents are physically present and the child has to identify the correct referent in ambiguous contexts (Quine, 1960; Snedeker & Gleitman, 2004). However, referents are not always present: caregivers can – and often do (Tomasello & Kruger, 1992) – talk about spatially and temporally displaced referents (e.g., the toy in the other room or the walk in the park that is about to happen), and these displaced scenarios can also provide learning opportunities. Experimental studies indicate that children learn from displaced contexts better (Tomasello & Kruger, 1992) or at least equally well than in joint attentional contexts (Tomasello et al., 1996; Tomasello & Barton, 1994), but little is known about *how* children can learn in displaced contexts. We propose that a close analysis of non-arbitrary multimodal strategies used by caregivers in face-to-face communication may provide us with important insight.

Perniss et al. (2017) asked whether caregivers modify iconic BSL signs more often when referents were displaced than when they were visually available. Caregivers were presented with sets of toys in one condition, and asked to imagine (the child was not present) talking to their child about the toys. In a second condition, the same caregivers had to imagine talking to their child about the toys, but without the toys present. They found that while caregivers used points more often to refer to the toys when they were present, they modified iconic signs more often (by enlargement, lengthening and repetition, as typical of child-directed language; Holzrichter & Meier, 2000; Pizer & Meier, 2011) when communicating about absent objects. Importantly, such differences between present and absent objects were not observed for less iconic signs, supporting the view that indeed these modifications are not just attention-grabbers. These results indicate instead that caregivers exploited the imagistic potential offered by iconicity as a resource particularly helpful in displaced contexts in helping the child map words to referents.

**Figure 2:**
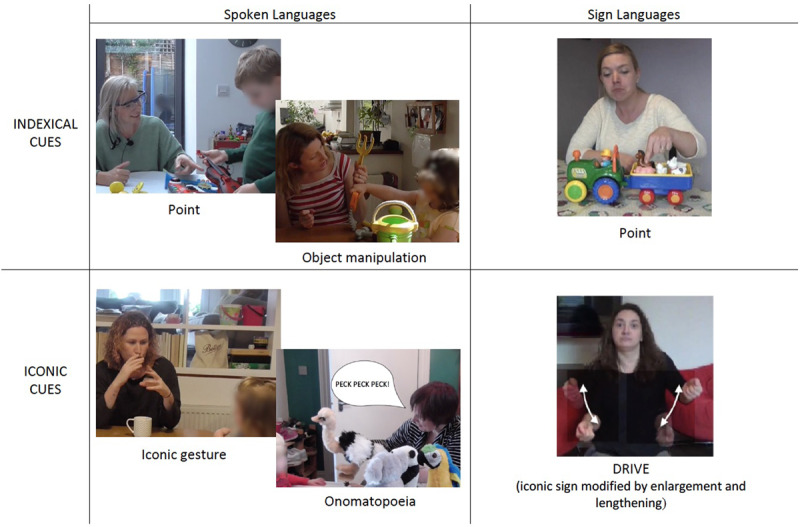
Examples of indexical cues (such as points and hand actions) and iconic cues (such as gestures, onomatopoeia and prosodic modulations of iconic signs) found in parental semi-naturalistic productions in spoken (Vigliocco et al., 2019) and sign languages (Perniss et al., 2017).

Using a similar design (but with the children present), Vigliocco et al. (2019) focused on child-directed spoken language, asking English speaking caregiver-child (2-3 years old) dyads to engage in conversation about toys both when they were present and absent. To better identify learning episodes, the toys were also divided into those known and unknown to the child. The authors analysed cues produced by the caregivers, coding indexical (points and object manipulations) and iconic cues (onomatopoeia and co-speech gestures) across different channels, demonstrating that these non-arbitrary multimodal behaviours are well represented in the input, accompanying almost 40% of caregiver utterances. Similarly to Perniss et al. (2017), they found that while indexical cues were overwhelmingly more common when the objects were present, onomatopoeia and iconic gestures were used more frequently in displaced contexts, and most often when the referent was unknown to the child. We illustrate examples of the different non-arbitrary cues found to constitute caregivers’ input in both sign and spoken languages in Figure 2.

By giving a more comprehensive picture of how language is acquired in the whole range of possible learning contexts, Perniss et al. (2017) and Vigliocco et al. (2019) go beyond the view of non-arbitrariness as a feature present or absent in the linguistic system, asking instead what do caregivers do when they use both systemic resources and multiple channels available in face-to-face communication. In the case of Perniss et al. (2017), we propose that this approach importantly overcomes a view inherited from a first scientific view on sign languages: that few signs can be considered effectively iconic, i.e., transparent (Bellugi & Klima, 1976; Klima & Bellugi, 1979), and that iconicity necessarily declines in a language in response to structural changes (Frishberg, 1975). This view, motivated by the initial need to recognise sign languages as proper linguistic systems, equivalent to spoken languages, led researchers to emphasize their structural similarities with spoken languages, minimizing their pictorial aspects (Kendon, 2012). However, several studies have shown that the so-called *frozen* iconicity of lexical signs can be modified during language use (Brennan, 1991, 1992; Cuxac, 2003; Johnston & Schembri, 1999; Russo, 2004), showing an online process that goes in the opposite direction with respect to the system-wide diachronic tendency for forms to become less iconic. In this way, iconicity in sign languages appears as a resource that, being well-incorporated into the phonological and grammatical constraints of the system, can be also retrieved and enriched in online language use.

Vigliocco et al. (2019) show that non-arbitrariness is pervasive in spoken languages during the communication between a caregiver and a child, as they find a noticeable presence of both iconic and indexical cues in caregivers’ input in both the vocal and the manual channel. Furthermore, the study shows that also in spoken languages different non-arbitrary cues are used by caregivers when they are most useful to children according to the context. Indexical cues – which signify the object by creating a visual link to it – are helpful when the referent is visually accessible: they can single it out in a cluttered visual scene. Iconic cues, on the other hand, represent the referent through a selection of its experiential properties that, even if absent from the environment, are brought into the communicative context. The study shows that iconicity is especially used when the label is unfamiliar to the child and the referred-to object is not immediately available in the environment. Here iconicity can bring to the “mind’s eye” properties of the referent. These results highlight the need to take into account both different modalities and different types of learning contexts; e.g., both joint attentional and displaced, to fully understand how children (and their caregivers) exploit non-arbitrary cues as a communicative resource.

## 5. Non-Arbitrariness in Language Processing

A role for context in language processing has long been recognized, with growing attention from researchers in the last decade or so (see reviews in Cai & Vigliocco, 2018; Meteyard & Vigliocco, 2018; Yee & Thompson-Schill, 2016). It is however the case that most current work still focuses on a single contextual factor. For example, researchers working with variants of the visual world paradigm (Tanenhaus et al., 1995) in which eye movements to depicted referents are recorded while subjects listen to speech, ignore the visual cues provided by the speaker (e.g., their gestures); those working on gestures ignore the physical context and those working on prosody often ignore all visual cues. This is done in order to secure experimental control. However, in this manner ecological validity is jeopardized (e.g., Hasson et al., 2018) and, crucially for our purposes here, visual processes based on indexical cues and imagistic processes based on iconic cues can be missed. Keeping this general issue in mind, let us briefly review some of the relevant literature.

Many studies have investigated how iconic gestures contribute to language comprehension and production. In language comprehension, iconic gestures can disambiguate lexical meaning of homonyms (Holle & Gunter, 2007), can support comprehension when the speech is degraded (Drijvers & Özyürek, 2017; Holle et al., 2010) and can provide further details useful in building situational models (e.g., if a listener hears a speaker say ‘and then I paid’ whilst making a writing gesture, the listener can understand that the speaker paid using a cheque; Cocks et al., 2018). Drijvers and Özyürek (2017) further showed that iconic gestures play a more important role than visible mouth patterns in supporting spoken comprehension in noise, but that both cues together can contribute to comprehension.

Gestures have also been shown to play an important role in production, for example by facilitating lexical retrieval (Hadar & Butterworth, 2009; Rauscher et al., 1996), or chunking information for verbal encoding (Alibali et al., 2000; Kita et al., 2017). Kita et al. (2007) showed that the syntactic organisation of manner and path information had an impact on the types of gestures participants produced, indicating that not only can speech and gesture affect each other, but that the semantic coordination between speech and gesture occurs online and is dependent on the linguistic context.

Iconic prosody has also been shown to contribute to meaning-making in online language use. In a series of studies, Shintel and colleagues (Shintel et al., 2006, 2014) investigated the role of prosody in both production and comprehension. Shintel, Nusbaum and Okrent (2006) showed English speaking adults animations of a dot moving in different directions (up or down) and at different speeds (fast or slow) going left or right. When participants were asked to describe the direction of the dot, participants showed changes in F0 that matched the up/down direction of the dot’s movement in the first case, and similar modulation in speech rate in the second case. Particularly in the second case, where the lexical cue does not match the prosodic modulation, the results suggest that participants convey meaning in prosody independent of the meaning conveyed lexically. Shintel, Anderson and Fenn (2014) tested whether prosodic modulations affect comprehension. They found that congruent prosody (e.g., high pitch conveying small size) allowed participants to identify referents more easily. Prosodic cues, like iconic gestures and points, contribute to meaning-making in both production and comprehension, sometimes supplying information independent of lexical content.

In the manual modality, just like in language development, the communicative context does not only provide iconic cues, but also indexical cues. A number of studies have analysed the relationship between pointing gestures and the use of demonstratives such as “this” and “that” (see Peeters & Özyürek, 2016 for a review). It has been shown that pointing is favoured over linguistic description to direct attention over small distances (Bangerter, 2004; Cooperrider, 2016), but conversely, linguistic description takes over for larger distances where pointing is less precise. Cooperrider (2016) also found that pointing could affect the type of demonstrative used, such that proximal demonstratives (e.g., “this”) were preferred when points were produced, and distal demonstratives (e.g., “that”) when points were absent. Taken together, these results suggest that speech and points form a combined system in face-to-face communication, used to effectively direct attention to specific locations or referents. Eye gaze can also provide a powerful visual cue to what is being talked about and facilitate processing (Holler et al., 2014).

The wide range of evidence concerning iconic and indexical communicative cues suggests that language as it is used in online, face-to-face interaction between both adults and children cannot be confined to a system of context-independent, linguistic components, nor can it be assigned only a grounding role, useful in the early stages of language learning. Rather, language being a dynamic, multimodal system situated in a given communicative and physical context, adaptively uses multiple arbitrary and non-arbitrary cues (arbitrary words and onomatopoeia available in the linguistic system, as well as iconic gesture, deixis and prosody) to contribute to context-dependent meaning making. This understanding of language as multimodal and dynamic in nature highlights the need to study different cues in combination, rather than as individual and somewhat unrelated elements of communication. A number of studies have made progress in this direction, highlighting how comprehension is modulated by multiple cues in interaction, such as gesture, eye gaze, mouth movements and prosodic modulations (Drijvers & Özyürek, 2017; Holler et al., 2014; Zhang et al., 2019). We propose that, going forward, we need language models that comprehensively examine the situated and multimodal nature of language, that account for non-arbitrary mappings from different sources, both in isolation and in combination with each other.

## 6. Non-Arbitrariness and Situated Language

We have shown in the previous sections that non-arbitrariness becomes clearly visible when we look at multimodal situated language learning and processing. In contrast, non-arbitrariness is subtle if we consider language only at the system-level. In fact, from the perspective of situated language, the extent of non-arbitrariness has not decreased to a negligible amount through evolution, as has been argued to be the case for iconicity in the linguistic system. In our corpus of semi-naturalistic language between 2-3 year old children and their caregivers, we see that approximately 39% of the utterances produced by caregivers contain indexical cues, and 17%^1^ iconic cues (Vigliocco et al., 2019). Thus, far from being negligible, non-arbitrary multimodal cues are pervasive in communication to young children. Why would this be the case? For iconicity, as already mentioned above, we argue that it can have an important role in providing imagistic information about the objects or events being discussed, helping in bringing back to mind the memory traces of an already experienced object or providing cues to build the conceptual knowledge relative to a completely new object (Vigliocco et al., 2019). A second important observation from our corpus is that while iconicity in speech (in the form of onomatopoeia) *decreases* significantly between 2 to 4 years of age, it does *increase* significantly in iconic gestures used by caregivers (Motamedi et al., 2020). This fact suggests that rather than disappearing, iconicity moves from the linguistic form, to leave room for a larger linguistic repertoire, to context-dependent recruitment of other modalities. Note here that in sign languages, the possibility to offload iconicity to the auditory channel is unavailable, which may explain why sign languages in general tend to have far more context-independent iconicity in the primary channel than spoken languages; i.e., meaningful form-meaning association in the linguistic system, for example at the phonological or lexical level. With respect to indexicality, we show that this strategy, already known to be used very early on by caregivers and children, is pervasive in joint-attentional communication through the use of different cues like pointing gestures as well as object manipulations which are able to anchor the message to the physical context increasing its referential transparency (Vigliocco et al., 2019).

For children and adults, non-arbitrariness in the communicative context is present beyond the negligeable remnants we see in linguistic forms. Although we do not have at the moment any estimate of how often multimodal cues would be used in language processing by adults, in the previous sections we presented abundant evidence for their presence and for their facilitatory function in processing. We argue that the multimodal communicative cues present in situated language processing serve the key function of supporting the development of an aligned situational model shared by the speaker/signer and the addressee. For the addressee, non-arbitrary cues produced by the speaker/signer support this function in at least two ways. First, by reducing potential ambiguity in the developing situational model. They provide direct visual links (with indexicality) and imagistic links (with iconicity) that can clarify and complement the primary channel; e.g., points can direct the attention to the referred-to object or a specific part of it, disambiguating the message; iconic gestures can provide information about the direction of motion in a 3D model that can be absent in speech; prosody and sound effects can replace words in providing information about speed of events, acoustic features of events, etc. Second and more broadly, they can reduce the cognitive effort required in generating situational models when the physical context does not provide any cue (when the language is displaced) and therefore the model needs to be generated internally. As displacement is a key feature of language (Hockett, 1960; Perniss & Vigliocco, 2014), it is not surprising that our communicative system would maintain features such as iconicity that can make displaced language easier to process.

For speakers and signers, multimodal non-arbitrary cues may also serve multiple functions. For example, for iconic gestures Kita et al. (2017) proposed the gesture-for-conceptualization hypothesis, according to which iconic gestures affect speakers’ cognitive processes in four different manners: they activate, manipulate, package, and explore spatio-motoric information for thinking and speaking. These four functions are shaped by gesture’s ability to schematize information. Thus, iconic gestures would serve self-oriented functions in addition to communicative functions. Onomatopoeia have been shown to be more resistant to brain damage than arbitrary words (Meteyard et al., 2015) possibly because retrieval of their phonological form can benefit from the support of non-linguistic information (see Vigliocco & Kita, 2006). Thus, multimodal iconicity in situated language could bring benefits not just to addresses but also speakers.

## 7. Conclusions

In this paper we provide a novel theoretical view on non-arbitrariness and its role in language which incorporates the language as a system perspective — according to which language is conceived as a system of symbols and rules that evolved at a population level — into the wider view of language as situated. Under this view, language — in its prime form i.e., face-to-face communication — is always embedded in a physical and communicative context in which speakers can and do use non-arbitrary multimodal cues, plausibly at least in part to support fluent and effortful production, and in which addressees can use these cues to learn language and to develop a situational model aligned with the speaker/signer’s. Crucially, the body of evidence we have reviewed suggests that cues beyond just speech and signs which are present in the face-to-face interactive context contribute to language acquisition and processing. Thus, language should be investigated as a multiplex and context dependent phenomenon if we seek a full account of how language has evolved, is learnt and is used in the real-world.

## Footnotes

This estimate does not include iconic modulations of prosody, hence it is bound to be under-estimating the actual incidence.
